# Central Obesity Is Associated with Variations in TSH and ACTH Levels among Euthyroid Obese Individuals

**DOI:** 10.1155/2022/3830380

**Published:** 2022-03-10

**Authors:** Difei Lu, Zhenfang Yuan, Ying Gao, Wei Liu, Junqing Zhang

**Affiliations:** Peking University First Hospital, Department of Endocrinology, Beijing, China

## Abstract

**Introduction:**

The interactions of central obesity and body composition with thyroid hormones and the hypothalamus-pituitary-adrenal (HPA) axis are unclear; both central obesity and body composition have an impact on energy homeostasis. Our study aimed to investigate the association between body composition and pituitary hormones, including the HPA axis and pituitary-thyroid axis, in a Chinese population of euthyroid overweight and obese individuals.

**Methods:**

This was a cross-sectional study. Overweight and obese patients who regularly visited the multidisciplinary team (MDT) for obesity at Peking University First Hospital were enrolled in the study. Thyroid function, morning serum ACTH and cortisol levels, thyroid peroxidase antibody (TPOAb), thyroglobulin antibody (TgAb), body composition, and metabolic indicators, including liver function and the lipid profile, were measured at the first visit. Statistical analysis was performed using SPSS version 21.0 (IBM, USA).

**Results:**

In total, 441 patients with overweight or obesity were enrolled (male/female, 123/318). Patients were assigned to four groups according to the thyroid-stimulating hormone (TSH) level stratified by quartiles, and increased body mass index (BMI) was revealed in the highest TSH quartile group (*p*=0.002). Hip circumference (HC) of patients in the highest TSH quartile group was significantly increased (*p*=0.021). Morning ACTH levels and fasting insulin levels were significantly elevated in patients in the highest TSH quartile group (*p*=0.027 for fasting insulin, *p* < 0.001 for ACTH). In the female subgroup, patients in the highest TSH quartile group showed increases in BMI (*p*=0.010), waist circumference (WC) (*p*=0.007), muscle mass of the lower extremities (*p*=0.020), fasting C-peptide (*p*=0.031), and ACTH (*p*=0.002). In the male subgroup, patients in the highest TSH quartile group exhibited higher BMI (*p*=0.017), HC (*p*=0.036), and ACTH (*p*=0.003). Among patients in the highest ACTH quartile group, there was an elevated proportion of males (*p*=0.003), and FT3 (*p*=0.005), fasting insulin (*p*=0.037), and cortisol (*p* < 0.001) levels were increased. Weight (*p* < 0.001), BMI (*p* < 0.001), WC (*p* < 0.001), HC (*p* < 0.001), muscle mass of the upper extremities (*p*=0.003), muscle mass of the lower extremities (*p*=0.005), and total muscle mass (*p*=0.003) were elevated in patients in the highest ACTH quartile group. HC was found to be an independent factor after adjustment for other confounders and was positively associated with the TSH level (*p*=0.004 for the regression model, *B* = 0.152, *p*=0.004).

**Conclusions:**

BMI is positively correlated with TSH and ACTH levels in both male and female obese individuals. The ACTH level was positively associated with male sex and increased BMI and muscle mass. Hip circumference was an independent factor that was positively related to TSH levels.

## 1. Introduction

At present, obesity is one of the most critical health risks. Central obesity is a major risk factor for dyslipidemia, cardiovascular disease, type 2 diabetes, and some subtypes of cancer [1]. Moreover, central obesity and body composition can be modulated by thyroid hormones and the hypothalamus-pituitary-adrenal (HPA) axis, which take part in the regulation of basal energy consumption [2]. Cushing's syndrome is a common cause of central obesity, indicating that serum cortisol levels are positively associated with central adiposity and alterations in body composition. The variations in serum adrenocorticotropic hormone (ACTH) and cortisol levels in overweight or obese individuals for whom Cushing's syndrome has been ruled out have not been fully explored.

T3 is an important regulator of energy expenditure and thermogenesis, as well as glucose and lipid metabolism; thus, it is logical that weight gain is often preceded by treatment of hypothyroidism [3]. Both subclinical and overt hypothyroidism are related to increased body mass index (BMI) and obesity, and weight loss is a classic manifestation of hyperthyroidism [4]. However, the association between thyroid function and body composition in euthyroid individuals is still under debate. Researchers suggest that serum-free *T*_4_ (FT4) is negatively related to BMI, and a thyroid-stimulating hormone (TSH) level that is slightly elevated or at the upper limit of the normal range has been observed in euthyroid overweight or obese individuals [5]. The TSH level has been demonstrated to be positively related to the risk of nonalcoholic fatty liver disease (NAFLD) [6], although these results have not been universally accepted by all researchers. Therefore, this study aimed to investigate the association of body composition and metabolic diseases with pituitary hormones, including the HPA axis and pituitary-thyroid axis, in a Chinese population of euthyroid overweight or obese individuals who visited the multidisciplinary outpatient clinic of Peking University First Hospital.

## 2. Methods

### 2.1. Settings

The multidisciplinary team (MDT) for obesity at Peking University First Hospital was launched on June 1^st^, 2015. Individuals aged 18–75 years with a BMI ≥24 kg/m^2^ were defined as overweight, and those with a BMI ≥28 kg/m^2^ were defined as obese. Patients who met the above criteria and were consecutively followed up in the obesity MDT from January 2016 to December 2020 were enrolled in the study. Thyroid function, morning serum ACTH and cortisol levels, thyroid peroxidase antibody (TPOAb) and thyroglobulin antibody (TgAb) were measured at the first visit. The thyroid function tests included TSH, FT4, free *T*_3_ (FT3), total *T*_4_ (T4) and total *T*_3_ (T3). Individuals diagnosed with Cushing's syndrome, Hashimoto's thyroiditis and overt hypothyroidism or hyperthyroidism (the exclusion criteria) were excluded at the first visit. Additionally, postsurgery patients with a past history of papillary thyroid carcinoma (PTC) were excluded.

A total of 516 patients finished the questionnaire and underwent a laboratory evaluation of thyroid function and morning cortisol and ACTH levels at the first visit. Additional patients were excluded as follows: 4 females were diagnosed with Graves' disease, 14 patients were diagnosed with PTC (male/female, 4/10), 47 patients were diagnosed with Hashimoto's thyroiditis (male/female, 3/44) and 10 patients were diagnosed with overt hypothyroidism requiring further levothyroxine replacement therapy (male/female, 1/9). Overall, 441 patients with overweight or obesity were enrolled after the first visit. Among these excluded patients, 123 individuals were male, and 318 were female.

### 2.2. Body and Laboratory Measurements

All participants underwent body measurements under fasting conditions, including body height (BH), body weight (BW), waist circumference (WC) and hip circumference (HC). BMI was calculated as body mass (kg)/height (m^2^). Male patients with a WC exceeding 90 cm or female patients with a WC over 85 cm were considered to have central obesity. Demographic data, including age, sex, smoking status, daily food intake and past medical history, which focused on whether the participant had type 2 diabetes, hypertension, hyperlipidemia, hyperuricemia, NAFLD, and polycystic ovary syndrome (PCOS), were collected using a questionnaire. A fasting venous blood sample was collected from each subject for the measurement of thyroid function, ACTH, cortisol, liver function, the lipid profile, HbA1c, fasting plasma glucose, plasma uric acid, and fasting plasma insulin. Body fat percentage (BF%), lean body mass (kg), skeletal muscle mass (kg), muscle mass of the upper extremities (kg), muscle mass of the lower extremities (kg), visceral fat area (cm^2^) and other body composition variables were evaluated using bioelectrical impedance analysis (InBody® 770 Medical Body Composition Analyzer, Biospace Co., Ltd, Korea).

### 2.3. Statistics

Statistical analysis was performed using SPSS version 21.0 (IBM, USA). Continuous variables are described as the mean ± standard deviation (SD), and categorical variables are described as percentages. Student's *t* test was used to compare within-group mean differences. The chi-square test for independence was used to compare the differences between categorical variables. To investigate the correlation between the exposures (obesity) and the outcomes (TSH), linear regression analysis was performed to investigate possible confounders for TSH, including age, sex, body composition and thyroid hormone, cortisol, insulin, C-peptide, glucose and lipid levels. A *p* value < 0.05 was considered indicative of statistical significance.

## 3. Results

### 3.1. The Distribution of Body Composition and Metabolic Indicators of Overweight and Obese Individuals according to TSH Quartiles

In total, 441 patients with overweight or obesity were enrolled (male/female, 123/318). The median TSH concentration in female subjects was higher than that in male subjects (male vs. female, 2.03 vs. 2.40 *μ*IU/ml, *p*=0.013), but FT3 and FT4 levels were decreased in female subjects compared with male subjects (FT3, male vs. female, 5.00 vs. 4.62 pmol/L, *p*=0.002; FT4, male vs. female, 16.99 vs. 15.12 pmol/L, *p*=0.024). The mean ACTH concentration, rather than cortisol level, was higher in the male group than in the female group (male vs. female, 34.56 vs. 26.86 pg/ml, *p*=0.001).

Patients were assigned to four groups according to TSH concentration stratified by quartiles, and anthropometric characteristics, body composition and metabolic indicators were compared among the four groups, as illustrated in Table 1. There was no significant difference in age distribution in the four groups or in the co-occurrence rate of other metabolic diseases, including dyslipidemia, diabetes mellitus (DM), hypertension, NAFLD, gout, hyperuricemia and PCOS. Females with overweight or obesity presented with higher TSH values (*p*=0.034). Increased BMI was revealed in the highest TSH quartile group (*p*=0.002). HC was significantly increased in patients in the highest TSH quartile group (*p*=0.021). The distribution of other body composition indicators, including weight, BF%, total muscle mass, muscle mass of the upper/lower extremities and basal metabolic rate (BMR), was nonsignificant in the TSH-stratified groups. Morning ACTH levels and fasting insulin levels were significantly increased in patients in the highest TSH quartile group (*p*=0.027 for fasting insulin, *p* < 0.001 for ACTH). Other indicators, including cortisol, fasting C-peptide, UA and transaminase levels, and the lipid profile, were comparable between groups.

### 3.2. The Distribution of Body Composition and Metabolic Indicators in Male or Female Subgroups of Overweight or Obese Individuals according to TSH Quartiles

In the female subgroup, there was an increased proportion of individuals with higher BMI, WC, muscle mass of the lower extremities, and fasting C-peptide and ACTH levels in the highest TSH quartile group (*p*=0.010 for BMI, *p*=0.007 for WC, *p*=0.020 for muscle mass of the lower extremities, *p*=0.031 for fasting C-peptide level, *p*=0.002 for ACTH level, Table 2). Hormones in the hypothalamic-pituitary-gonadal axis exhibited a nonsignificant distribution across the TSH-stratified groups. In the male subgroup, patients in the highest TSH quartile group exhibited higher BMI, HC and ACTH levels (*p*=0.017 for BMI, *p*=0.036 for HC, *p*=0.003 for ACTH, Table 3).

### 3.3. The Distribution of Pituitary Hormones in the Overweight and Obesity Subgroups

Among the 441 patients enrolled, 55 patients were overweight, and 386 patients were obese. No significant difference in TSH or cortisol levels was found (TSH, overweight vs. obesity, 2.49 ± 1.68 vs. 2.67 ± 1.84 *μ*IU/ml, *p*=0.500; cortisol, overweight vs. obesity, 13.9 ± 4.8 vs. 14.3 ± 5.5 *μ*g/dl, *p*=0.687). The ACTH level was significantly higher in the obesity group than in the overweight group (overweight vs. obesity, 22.7 ± 9.3 vs. 30.0 ± 18.1 pg/ml, *p* < 0.001).

We further explored the distribution of body composition and metabolic indicators according to TSH quartiles in the overweight and obesity subgroups. In the obesity subgroup, there was an increased proportion of individuals with higher BMI, HC, and fasting insulin and ACTH levels in the highest TSH quartile group (*p*=0.004 for BMI, *p*=0.045 for HC, *p*=0.045 for fasting insulin level, *p* < 0.001 for ACTH level). No significant difference in the distribution of body composition, ACTH level, or metabolic indicators was discovered according to TSH quartiles in the overweight subgroup.

### 3.4. The Distribution of Body Composition and Metabolic Indicators in Overweight and Obese Individuals according to ACTH Quartiles

Patients were assigned to four groups according to ACTH levels stratified by quartiles, and thyroid function, body composition, and metabolic indicators were compared across the four groups (Table 4). There was no significant difference in the metabolic indicators across the groups. Among the patients in the highest ACTH quartile group, there was an elevated proportion of males (*p*=0.003). Moreover, patients in the highest ACTH quartile had increased hormone levels, including FT3; fasting insulin; and cortisol levels (*p*=0.005 for FT3, *p*=0.037 for fasting insulin, *p* < 0.001 for cortisol). Body composition indicators, including body weight, BMI, WC, HC, muscle mass of the upper extremities, muscle mass of the lower extremities, and total muscle mass, were elevated in patients in the highest ACTH quartile group (*p* < 0.001 for weight, *p* < 0.001 for BMI, *p* < 0.001 for WC, *p* < 0.001 for HC, *p*=0.003 for muscle mass of the upper extremities, *p*=0.005 for muscle mass of the lower extremities, *p*=0.003 for muscle mass).

### 3.5. Correlation Analysis of Confounders of TSH in Overweight and Obese Individuals

Overall, BMI, WC, HC, BF%, and levels of fasting insulin, fasting C-peptide, ACTH, and low-density lipoprotein (LDL) were positively correlated with TSH levels (*p*=0.001 for BMI, *p*=0.005 for WC, *p*=0.006 for HC, *p* < 0.001 for BF%, *p*=0.001 for fasting insulin level, *p*=0.008 for fasting C-peptide level, *p*=0.004 for ACTH level and *p*=0.01 for LDL level, Table 5). However, hip circumference was found to be an independent factor after adjustment for FT3, FT4, T3, T4, body weight, BMI, waist circumference, fat percentage, skeletal muscle mass, basal metabolic rate, and fasting insulin level, and it was positively associated with TSH level (*p*=0.004 for the regression model, *B* = 0.152, *p*=0.004).

## 4. Discussion

This study was carried out in a Chinese population of euthyroid individuals diagnosed with overweight or obesity and aimed to investigate the slight variation in TSH and ACTH concentrations within the normal range in this population. Our findings show that independent of age, BMI, muscle mass, fat percentage, and serum insulin levels, the subjects with excess weight, especially elevated hip circumference, had a higher possibility of increased circulating TSH levels, which is accompanied by an increased ACTH level.

Previous studies have explored the intriguing association between thyroid function and obesity. A population study was conducted with euthyroid obese individuals and revealed that the FT4 level was inversely related to BMI, insulin resistance and triglycerides, while the TSH concentration was positively associated with BMI and total cholesterol levels [7]. The TSH level could deviate along with body mass. Researchers conducted a 4-week lifestyle intervention program, and TSH levels were reduced in euthyroid obese patients after weight reduction greater than 5% [8]. Additionally, a population-based longitudinal study revealed the possibility of weight gain in individuals with higher TSH values during a 5-year follow-up [9]. In line with a previous study, our study discovered that both the male and female subgroups of overweight and obese patients in the highest TSH quartile, which was over 3.15 *μ*IU/ml, had an increased BMI, although FT4 showed no significant correlation with BMI.

In addition to thyroid function, central obesity is associated with the occurrence of thyroid nodules, as revealed in a cross-sectional study in China [10], which indicated that patients with central obesity were at 1.62-fold higher risk of thyroid nodules than those with a normal waist circumference. Data on thyroid nodules were neither collected nor analyzed in our study, but 14 patients with existing PTC were excluded from the study population. One explanation for the relatively high prevalence of PTC (14/516) in our study population is the fact that TSH is a positive risk factor for PTC [11], while TSH was elevated in overweight and obese individuals according to our findings.

This study revealed that hip circumference was an independent factor that was positively related to TSH levels. In terms of body composition, TSH was proven to be positively associated with waist circumference in overweight and obese women [12]. A case-control study found that T3 and T4 levels were positively correlated with body fat percentage in normal-weight women with a high body fat percentage [13]. Waist circumference is a primary indicator of central obesity, which is a critical risk factor for metabolic diseases, including DM, and increased hip circumference is commonly seen among individuals with the so-called pear body shape. Our study informs endocrinologists that increased attention needs to be given to obese patients with a pear body shape regarding screening for thyroid function.

There was no significant correlation between body fat percentage and TSH level in our study, although researchers have found that in healthy individuals, subcutaneous fat was positively correlated with TSH levels [14]. A similar result was found in visceral adipose tissue volumes associated with high TSH values [15]. Comparisons with normal-weight subjects could further be performed in our population to investigate the possible correlation of thyroid function and BF%.

A previous study discovered a positive linkage between muscle mass and the FT3/FT4 ratio, suggesting that increased skeletal muscle mass in overweight and obese individuals results in increased conversion from T4 to T3 [16, 17]. In our study population, muscle mass of the lower extremities was positively associated with TSH levels in both the male and female subgroups, although it no longer appeared significant in the regression analysis after adjustment for BMI.

One possible explanation for the alteration of the TSH levels in obese patients was the increased leptin level, which can affect thyroid deiodinase activity and accelerate the conversion of T4 to T3 [18]. Another possible mechanism involves TSH receptors. Despite the elevated circulating TSH levels, fewer TSH receptors are expressed on adipocytes of obese patients than on those of lean individuals, which further increases plasma TSH levels and results in peripheral thyroid hormone resistance [19].

Studies on the interaction between the HPA axis and obesity revealed increased cortisol secretion in obese patients accompanied by elevated ACTH levels [20, 21], which was similar to the findings of our study. Although the HPA axis was stimulated in obese individuals, the responsiveness of the adrenal gland to ACTH was diminished [22]. In our study population, increased ACTH levels were associated with male individuals with larger body weight and muscle mass, as well as elevated FT3 levels. As mentioned above, skeletal muscle mass was related to the increased conversion of T4 to T3 [16, 17]. Male sex did not have an independent impact on the ACTH level, which was proven in a clinical trial assessing the ACTH stimulation test in the evaluation of the hypothalamo-pituitary-adrenal axis in healthy adults [23]. In addition, it was demonstrated that both leg and arm exercise could stimulate the HPA axis immediately after exercise [24]. However, no study has provided evidence on the effect of ACTH concentration on muscle mass.

It was postulated that the adrenal autonomic response might be weakened along with an increase in accumulated adipose tissue. TSH and ACTH are fragments derived from proopiomelanocortin (POMC), and both play critical roles in energy homeostasis. In obese individuals, elevated TSH and ACTH levels with relatively unchanged thyroid hormone and cortisol levels may be explained by a diminished response to epistatic hormones due to accumulated adipose tissue. This hypothesis and the interplay of POMC peptides and excess adipose tissue need to be investigated in further studies using rodent models.

Our study is a retrospective study, and the main limitation is that our findings could reveal only correlations instead of cause and effect. The study population comprised overweight and obese individuals, and there was a lack of subjects with normal BMI for comparison.

In conclusion, BMI is positively correlated with TSH levels in both male and female obese individuals, and an increase in ACTH levels was also observed. The ACTH level was positively associated with male sex, increased BMI and muscle mass. Hip circumference was an independent factor that was positively related to TSH levels.

## Figures and Tables

**Table 1:** The distribution of demographic data and metabolic indicators across the TSH quartiles in overweight or obese individuals.

TSH quartiles (*μ*IU/ml)		≤1.55	1.56–2.24	2.25–3.14	≥3.15	*p* value
Age (year)	*N*	112	108	109	110	0.621
	Mean ± SD	35.1 ± 12.3	36.9 ± 14.2	34.7 ± 12.1	35.6 ± 12.3	

Sex (male/female)	*N*	36/76	40/72	24/83	23/87	**0.034**

Dyslipidemia	*N*, %	50, 44.6%	50, 44.6%	42, 39.3%	53, 48.2%	0.618

DM	*N*, %	35, 31.3%	32, 28.6%	31, 29.0%	35, 31.8%	0.938

HT	*N*, %	29, 25.9%	36, 32.1%	23, 21.5%	36, 32.7%	0.199

NAFLD	*N*, %	83, 74.1%	94, 83.9%	79, 73.8%	82, 74.5%	0.215

Gout	*N*, %	3, 2.7%	8, 7.1%	5, 4.7%	2, 1.8%	0.189

Hyperuricemia	*N*, %	34, 30.4%	31, 27.7%	29, 27.1%	27, 24.5%	0.813

PCOS	*N*, %	12, 15.8%	11, 15.3%	18, 21.7%	14, 16.1%	0.675

FT3 (pmol/L)	*N*	54	54	48	63	0.521
	Mean ± SD	4.68 ± 0.93	4.58 ± 0.67	4.77 ± 0.89	4.78 ± 0.59	

FT4 (pmol/L)	*N*	53	51	45	64	0.183
	Mean ± SD	15.92 ± 2.68	15.20 ± 2.00	15.03 ± 2.10	15.11 ± 2.38	

TT3 (nmol/L)	*N*	104	99	95	99	0.408
	Mean ± SD	1.73 ± 0.37	1.74 ± 0.29	1.77 ± 0.30	1.80 ± 0.31	

TT4 (nmol/L)	*N*	103	99	95	99	0.473
	Mean ± SD	104.6 ± 20.6	101.2 ± 19.8	102.1 ± 19.5	105.1 ± 21.7	

Weight (kg)	*N*	112	108	110	110	0.436
	Mean ± SD	98.3 ± 8.9	92.4 ± 1.9	90.8 ± 1.8	104.8 ± 9.5	

BMI (kg/m^2^)	*N*	112	108	110	110	**0.002**
	Mean ± SD	32.4 ± 4.6	33.5 ± 5.7	32.8 ± 4.5	34.9 ± 6.1	

WC (cm)	*N*	111	107	110	109	0.090
	Mean ± SD	101.0 ± 13.0	102.1 ± 14.5	101.7 ± 12.5	105.3 ± 13.9	

HC (cm)	*N*	111	107	110	109	**0.021**
	Mean ± SD	110.9 ± 9.2	112.4 ± 12.2	111.5 ± 9.4	115.2 ± 12.8	

BF (%)	*N*	111	108	110	110	0.151
	Mean ± SD	41.5 ± 11.6	41.7 ± 6.4	41.5 ± 5.7	44.1 ± 13.1	

Muscle mass of upper extremities (kg)	*N*	92	98	95	97	0.722
	Mean ± SD	5.74 ± 1.65	5.81 ± 1.53	5.71 ± 1.47	5.95 ± 1.63	

Muscle mass of lower extremities (kg)	*N*	92	98	95	97	0.636
	Mean ± SD	16.17 ± 3.94	16.21 ± 3.31	16.11 ± 3.56	16.72 ± 3.71	

Muscle mass (kg)	*N*	93	99	95	97	0.751
	Mean ± SD	29.07 ± 7.34	29.05 ± 6.50	28.64 ± 6.27	29.71 ± 7.01	

BMR (kCal)	*N*	109	105	108	107	0.766
	Mean ± SD	1548.6 ± 307.0	1539.0 ± 297.5	1539.7 ± 274.5	1576.4 ± 290.8	

Daily calorie intake (kCal)	*N*	38	29	31	33	0.626
	Mean ± SD	2256.3 ± 158.3	2199.3 ± 144.0	2147.5 ± 113.6	2018.9 ± 109.2	

Fasting insulin (*μ*IU/ml)	*N*	68	83	86	73	**0.027**
	Mean ± SD	23.81 ± 1.92	24.73 ± 2.15	24.17 ± 1.62	32.88 ± 3.65	

Fasting C-peptide (ng/ml)	*N*	65	78	77	64	0.053
	Mean ± SD	3.70 ± 1.45	3.47 ± 1.56	3.79 ± 1.74	4.23 ± 1.82	

Cortisol (*μ*g/dl)	*N*	76	81	86	76	0.485
	Mean ± SD	13.94 ± 5.62	14.34 ± 5.80	13.80 ± 4.98	15.03 ± 5.15	

ACTH (pg/ml)	*N*	75	81	82	78	**<0.001**
	Mean ± SD	26.55 ± 16.83	27.03 ± 13.53	26.57 ± 13.16	36.74 ± 23.10	

HbA1c (%)	*N*	58	58	70	57	0.780
	Mean ± SD	6.60 ± 1.49	6.61 ± 1.74	6.36 ± 1.38	6.51 ± 1.50	

ALT (IU/L)	*N*	85	85	85	77	0.806
	Mean ± SD	45.36 ± 4.28	44.51 ± 3.72	48.28 ± 5.93	50.59 ± 5.04	

AST (IU/L)	*N*	84	82	86	76	0.422
	Mean ± SD	28.98 ± 2.24	27.12 ± 1.68	31.12 ± 2.87	32.59 ± 2.81	

UA (*μ*mol/L)	*N*	83	83	84	77	0.532
	Mean ± SD	397.9 ± 115.2	387.9 ± 112.8	372.3 ± 122.0	381.3 ± 108.4	

TG (mmol/L)	*N*	84	86	85	75	0.287
	Mean ± SD	2.18 ± 1.69	1.91 ± 1.34	1.82 ± 0.97	2.11 ± 1.38	

TCHO (mmol/L)	*N*	83	84	84	74	0.332
	Mean ± SD	4.87 ± 0.99	4.73 ± 1.08	4.75 ± 0.93	5.02 ± 1.40	

HDL (mmol/L)	*N*	84	86	83	75	0.938
	Mean ± SD	1.13 ± 0.23	1.11 ± 0.22	1.11 ± 0.37	1.11 ± 0.22	

LDL (mmol/L)	*N*	84	86	83	74	0.245
	Mean ± SD	3.01 ± 0.73	3.01 ± 0.80	2.96 ± 0.80	3.22 ± 1.05	

DM, diabetes mellitus; HT, hypertension; NAFLD, nonalcoholic fatty liver disease; PCOS, polycystic ovary syndrome; BMR, basal metabolic rate; BMI, body mass index; WC, waist circumference; HC, hip circumference; BF, body fat percentage; ACTH, adrenocorticotropic hormone; ALT, alanine aminotransferase; AST, glutamic oxaloacetic transaminase; UA, uric acid; TG, triglyceride; TCHO, total cholesterol; HDL, high-density lipoprotein; LDL, low-density lipoprotein.

**Table 2:** The distribution of demographic data and metabolic indicators across the TSH quartiles in the female subgroup.

TSH quartiles (*μ*IU/ml)		≤1.55	1.56–2.24	2.25–3.14	≥3.15	*p* value
Age (year)	*N*	76	72	83	86	0.808
	Mean ± SD	36.5 ± 12.9	38.1 ± 14.4	36.3 ± 12.2	37.3 ± 12.6	

FT3 (pmol/L)	*N*	42	38	38	52	0.599
	Mean ± SD	4.54 ± 0.93	4.54 ± 0.66	4.72 ± 0.87	4.66 ± 0.50	

FT4 (pmol/L)	*N*	41	36	36	53	0.166
	Mean ± SD	15.69 ± 2.66	15.31 ± 2.09	14.93 ± 2.13	14.70 ± 2.03	

TT3 (nmol/L)	*N*	69	66	68	76	0.720
	Mean ± SD	1.72 ± 0.41	1.73 ± 0.28	1.75 ± 0.32	1.78 ± 0.32	

TT4 (nmol/L)	*N*	68	66	68	99	0.460
	Mean ± SD	107.3 ± 20.1	102.9 ± 19.8	102.4 ± 18.8	105.2 ± 21.4	

Weight (kg)	*N*	76	72	83	86	0.150
	Mean ± SD	82.4 ± 11.9	85.9 ± 17.2	84.9 ± 13.9	101.6 ± 12.1	

BMI (kg/m^2^)	*N*	76	72	83	86	**0.010**
	Mean ± SD	31.5 ± 4.1	32.9 ± 5.9	32.0 ± 4.2	34.1 ± 6.3	

WC (cm)	*N*	76	71	83	85	**0.007**
	Mean ± SD	96.1 ± 10.9	97.4 ± 11.9	98.0 ± 10.4	102.1 ± 13.2	

HC (cm)	*N*	76	71	83	85	0.082
	Mean ± SD	109.4 ± 8.8	111.5 ± 11.9	110.2 ± 9.0	113.5 ± 12.9	

BF (%)	*N*	76	72	83	86	0.119
	Mean ± SD	42.2 ± 5.3	44.4 ± 5.4	42.8 ± 5.2	45.2 ± 14.3	

Muscle mass of upper extremities (kg)	*N*	65	65	73	75	0.056
	Mean ± SD	4.91 ± 0.79	4.98 ± 0.98	5.09 ± 0.92	5.30 ± 0.92	

Muscle mass of lower extremities (kg)	*N*	65	65	73	75	**0.020**
	Mean ± SD	14.23 ± 1.88	14.42 ± 2.11	14.63 ± 2.21	15.30 ± 2.33	

Muscle mass (kg)	*N*	66	66	73	75	0.109
	Mean ± SD	25.56 ± 4.01	25.45 ± 3.98	25.97 ± 3.85	26.91 ± 3.94	

BMR (kCal)	*N*	76	70	82	84	**0.050**
	Mean ± SD	1417.4 ± 214.2	1400.3 ± 186.3	1431.8 ± 181.4	1484.6 ± 214.7	

Fasting insulin (*μ*IU/ml)	*N*	42	56	64	55	0.080
	Mean ± SD	24.46 ± 2.72	23.29 ± 2.57	22.62 ± 1.59	32.38 ± 4.64	

Fasting C-peptide (ng/ml)	*N*	39	53	54	48	**0.031**
	Mean ± SD	3.44 ± 1.45	3.35 ± 1.64	3.45 ± 1.26	4.20 ± 1.94	

Cortisol (*μ*g/dl)	*N*	48	54	63	59	0.168
	Mean ± SD	12.89 ± 4.57	13.53 ± 3.95	14.07 ± 4.71	14.84 ± 5.16	

ACTH (pg/ml)	*N*	47	55	60	60	**0.002**
	Mean ± SD	22.94 ± 11.83	24.94 ± 12.64	25.45 ± 11.81	33.32 ± 21.75	

HbA1c (%)	*N*	38	36	54	44	0.995
	Mean ± SD	6.39 ± 1.38	6.34 ± 1.18	6.36 ± 1.45	6.41 ± 1.31	

UA (*μ*mol/L)	*N*	54	56	61	58	0.708
	Mean ± SD	364.6 ± 12.4	359.5 ± 14.2	347.5 ± 12.6	365.9 ± 10.8	

Progesterone (ng/ml)	*N*	25	33	39	33	0.809
	Mean ± SD	1.87 ± 0.78	2.54 ± 0.89	3.07 ± 0.83	2.58 ± 0.78	

Testosterone (ng/ml)	*N*	24	33	40	34	0.227
	Mean ± SD	1.31 ± 0.42	0.67 ± 0.06	0.73 ± 0.08	1.81 ± 0.82	

Estradiol (pg/ml)	*N*	26	34	39	34	0.625
	Mean ± SD	82.85 ± 15.08	80.19 ± 10.91	103.79 ± 19.29	80.32 ± 14.33	

FSH (mIU/ml)	*N*	26	34	40	36	0.087
	Mean ± SD	6.16 ± 0.68	12.75 ± 3.03	7.59 ± 1.46	7.39 ± 1.41	

LH (mIU/ml)	*N*	26	36	40	36	0.192
	Mean ± SD	12.63 ± 3.59	9.32 ± 1.21	9.09 ± 1.26	6.94 ± 0.88	

BMR, basal metabolic rate; BMI, body mass index; WC, waist circumference; HC, hip circumference; BF, body fat percentage; ACTH, adrenocorticotropic hormone; UA, uric acid; FSH, follicle-stimulating hormone; LH, luteinizing hormone.

**Table 3:** The distribution of demographic data and metabolic indicators across the TSH quartiles in the male subgroup.

TSH quartiles (*μ*IU/ml)		≤1.55	1.56–2.24	2.25–3.14	≥3.15	*p* value
Age (year)	*N*	36	40	23	23	0.219
	Mean ± SD	32.2 ± 10.5	34.3 ± 13.3	28.7 ± 10.3	29.7 ± 9.1	

FT3 (pmol/L)	*N*	12	18	8	11	0.207
	Mean ± SD	5.20 ± 0.75	4.76 ± 0.81	4.81 ± 0.87	5.32 ± 0.71	

FT4 (pmol/L)	*N*	12	17	7	11	0.063
	Mean ± SD	16.66 ± 2.73	14.77 ± 1.77	16.04 ± 1.67	17.08 ± 3.03	

TT3 (nmol/L)	*N*	35	37	23	23	0.365
	Mean ± SD	1.74 ± 0.29	1.77 ± 0.32	1.84 ± 0.34	1.86 ± 0.28	

TT4 (nmol/L)	*N*	35	37	23	23	0.390
	Mean ± SD	99.4 ± 20.7	96.5 ± 19.5	104.2 ± 21.3	104.6 ± 23.2	

Weight (kg)	*N*	36	40	24	23	0.594
	Mean ± SD	103.7 ± 26.8	104.6 ± 19.1	109.8 ± 19.3	117.8 ± 17.2	

BMI (kg/m^2^)	*N*	36	40	24	23	**0.017**
	Mean ± SD	34.4 ± 5.1	34.6 ± 5.2	35.3 ± 4.7	38.3 ± 3.9	

WC (cm)	*N*	35	40	24	23	0.185
	Mean ± SD	111.7 ± 10.5	110.8 ± 14.6	113.3 ± 12.0	117.5 ± 9.1	

HC (cm)	*N*	35	40	24	23	**0.036**
	Mean ± SD	114.3 ± 9.2	114.1 ± 12.1	115.8 ± 10.2	121.8 ± 10.4	

BF (%)	*N*	35	40	24	23	0.566
	Mean ± SD	39.8 ± 3.25	36.5 ± 5.1	38.3 ± 5.5	39.8 ± 5.9	

Muscle mass of upper extremities (kg)	*N*	27	36	20	21	0.075
	Mean ± SD	7.76 ± 1.45	7.41 ± 1.04	7.74 ± 1.07	8.32 ± 1.40	

Muscle mass of lower extremities (kg)	*N*	27	36	20	21	0.053
	Mean ± SD	20.82 ± 3.71	19.67 ± 2.33	21.06 ± 2.72	21.88 ± 3.22	

Muscle mass (kg)	*N*	27	36	20	21	0.093
	Mean ± SD	37.65 ± 6.52	36.08 ± 4.33	37.39 ± 4.58	39.89 ± 6.24	

BMR (kCal)	*N*	33	39	23	22	0.331
	Mean ± SD	1850.7 ± 274.6	1806.6 ± 277.8	1885.5 ± 257.6	1935.9 ± 271.4	

Fasting insulin (*μ*IU/ml)	*N*	26	29	21	17	0.194
	Mean ± SD	22.78 ± 2.48	28.12 ± 3.66	27.91 ± 4.46	35.18 ± 4.74	

Fasting C-peptide (ng/ml)	*N*	26	27	21	16	0.471
	Mean ± SD	4.10 ± 1.39	3.81 ± 1.33	4.57 ± 2.49	4.34 ± 1.42	

Cortisol (*μ*g/dl)	*N*	28	29	22	16	0.448
	Mean ± SD	15.74 ± 6.79	15.85 ± 8.06	13.09 ± 5.62	15.68 ± 5.39	

ACTH (pg/ml)	*N*	28	28	21	17	**0.003**
	Mean ± SD	32.61 ± 21.84	32.36 ± 14.44	27.54 ± 16.05	50.10 ± 23.67	

HbA1c (%)	*N*	20	24	14	13	0.648
	Mean ± SD	7.01 ± 1.65	7.05 ± 2.30	6.29 ± 1.04	6.88 ± 2.02	

UA (*μ*mol/L)	*N*	29	31	20	18	0.862
	Mean ± SD	459.9 ± 23.2	435.2 ± 20.0	451.5 ± 33.6	431.1 ± 38.2	

Testosterone (ng/ml)	*N*	8	19	13	7	0.474
	Mean ± SD	3.84 ± 1.22	3.60 ± 1.76	2.96 ± 1.21	3.16 ± 0.75	

BMR, basal metabolic rate; BMI, body mass index; WC, waist circumference; HC, hip circumference; BF, body fat percentage; ACTH, adrenocorticotropic hormone; UA, uric acid.

**Table 4:** The distribution of demographic data and metabolic indicators across the ACTH quartiles in overweight or obese individuals.

ACTH quartiles (pg/ml)		≤17.31	17.36–25.24	25.26–37.02	>37.05	*p* value
Age (year)	*N*	80	79	79	79	0.466
	Mean ± SD	35.1 ± 11.6	32.1 ± 12.6	34.0 ± 11.2	33.5 ± 12.9	

Sex (male/female)	*N*	15/65	25/54	19/60	35/44	**0.003**

TSH (*μ*IU/ml)	*N*	79	79	79	79	0.206
	Mean ± SD	2.41 ± 1.56	2.62 ± 2.24	2.73 ± 1.42	3.04 ± 2.18	

FT3 (pmol/L)	*N*	29	33	33	39	**0.005**
	Mean ± SD	4.56 ± 0.86	4.53 ± 0.76	4.98 ± 0.49	5.02 ± 0.69	

FT4 (pmol/L)	*N*	27	30	35	38	0.456
	Mean ± SD	14.89 ± 2.58	15.44 ± 2.50	15.76 ± 1.87	15.24 ± 1.83	

TT3 (nmol/L)	*N*	71	70	75	76	0.277
	Mean ± SD	1.73 ± 0.31	1.80 ± 0.37	1.80 ± 0.32	1.83 ± 0.28	

TT4 (nmol/L)	*N*	71	70	75	76	0.479
	Mean ± SD	101.8 ± 20.0	104.6 ± 21.6	104.6 ± 21.0	100.2 ± 19.0	

Weight (kg)	*N*	80	79	79	79	**<0.001**
	Mean ± SD	88.3 ± 16.3	94.1 ± 18.1	89.3 ± 17.5	101.6 ± 21.1	

BMI (kg/m^2^)	*N*	80	79	79	79	**<0.001**
	Mean ± SD	32.6 ± 4.2	33.8 ± 5.7	32.6 ± 5.1	35.8 ± 5.4	

WC (cm)	*N*	80	79	79	79	**<0.001**
	Mean ± SD	100.2 ± 11.5	103.6 ± 13.0	99.3 ± 13.3	109.7 ± 13.9	

HC (cm)	*N*	80	79	79	79	**<0.001**
	Mean ± SD	110.2 ± 9.1	114.2 ± 12.6	110.6 ± 9.2	117.0 ± 11.8	

BF (%)	*N*	80	78	79	79	0.553
	Mean ± SD	42.4 ± 5.7	43.4 ± 5.5	41.3 ± 5.7	42.1 ± 6.7	

Muscle mass of upper extremities (kg)	*N*	71	65	72	64	**0.003**
	Mean ± SD	5.52 ± 1.49	5.92 ± 1.44	5.69 ± 1.49	6.46 ± 1.70	

Muscle mass of lower extremities (kg)	*N*	71	65	72	64	**0.006**
	Mean ± SD	15.71 ± 3.38	16.72 ± 3.30	16.12 ± 3.51	17.77 ± 4.02	

Muscle mass (kg)	*N*	72	66	72	64	**0.005**
	Mean ± SD	27.81 ± 6.38	29.72 ± 6.19	28.72 ± 6.39	31.77 ± 7.43	

Fasting insulin (*μ*IU/ml)	*N*	64	72	72	71	**0.037**
	Mean ± SD	26.81 ± 2.68	26.04 ± 2.27	22.04 ± 1.71	32.47 ± 3.38	

Fasting C-peptide (ng/ml)	*N*	57	68	69	66	0.077
	Mean ± SD	3.70 ± 1.81	3.89 ± 1.79	3.48 ± 1.31	4.20 ± 1.68	

Cortisol (*μ*g/dl)	*N*	80	78	78	75	**<0.001**
	Mean ± SD	11.61 ± 4.95	12.62 ± 3.68	15.13 ± 4.77	17.40 ± 5.53	

HbA1c (%)	*N*	44	49	52	53	0.410
	Mean ± SD	6.30 ± 1.13	6.60 ± 1.67	6.82 ± 2.02	6.45 ± 1.33	

ALT (IU/L)	*N*	63	67	64	67	0.582
	Mean ± SD	39.95 ± 4.64	49.68 ± 7.45	49.20 ± 5.31	47.92 ± 4.17	

AST (IU/L)	*N*	62	67	63	66	0.556
	Mean ± SD	26.28 ± 2.25	28.92 ± 2.88	31.33 ± 3.25	31.16 ± 2.71	

TG (mmol/L)	*N*	64	68	66	65	0.145
	Mean ± SD	1.76 ± 1.22	1.89 ± 1.31	2.19 ± 1.47	2.26 ± 1.70	

TCHO (mmol/L)	*N*	63	67	65	64	0.375
	Mean ± SD	4.73 ± 0.84	4.65 ± 1.03	4.94 ± 0.98	4.91 ± 1.50	

HDL (mmol/L)	*N*	63	68	66	65	0.521
	Mean ± SD	1.09 ± 0.23	1.13 ± 0.23	1.12 ± 0.38	1.07 ± 0.21	

LDL (mmol/L)	N	63	68	66	64	0.315
	Mean ± SD	2.93 ± 0.75	2.91 ± 0.73	3.15 ± 0.76	3.05 ± 1.11	

BMI, body mass index; WC, waist circumference; HC, hip circumference; BF, body fat percentage; ACTH, adrenocorticotropic hormone; TSH, thyroid-stimulating hormone; ALT, alanine aminotransferase; AST, glutamic oxaloacetic transaminase; UA, uric acid; TG, triglyceride; TCHO, total cholesterol; HDL, high-density lipoprotein; LDL, low-density lipoprotein.

**Table 5:** Correlation of demographic characteristics, body composition, and metabolic indicators with TSH in overweight and obese individuals.

	TSH (*μ*IU/ml)		
	*n*	Pearson correlation coefficient	*p* value
Age (year)	439	−0.003	0.955
Weight (kg)	440	0.013	0.790
BMI (kg/m^2^)	440	0.162	**0.001**
WC (cm)	437	0.135	**0.005**
HC (cm)	437	0.130	**0.006**
BF (%)	439	0.297	**<0.001**
Muscle mass (kg)	384	0.012	0.810
BMR (kCal)	429	0.016	0.734
Fasting insulin (*μ*IU/ml)	310	0.183	**0.001**
Fasting C-peptide (ng/ml)	284	0.157	**0.008**
Cortisol (*μ*g/dl)	319	0.020	0.725
ACTH (pg/ml)	316	0.164	**0.004**
ALT (IU/L)	332	0.065	0.235
AST (IU/L)	328	0.088	0.113
UA (*μ*mol/L)	327	−0.020	0.718
TG (mmol/L)	330	0.009	0.871
TCHO (mmol/L)	325	0.104	0.061
HDL (mmol/L)	328	−0.026	0.643
LDL (mmol/L)	327	0.141	**0.010**

BMR, basal metabolic rate; BMI, body mass index; WC, waist circumference; HC, hip circumference; BF, body fat percentage; ACTH, adrenocorticotropic hormone; ALT, alanine aminotransferase; AST, glutamic oxaloacetic transaminase; UA, uric acid; TG, triglyceride; TCHO, total cholesterol; HDL, high-density lipoprotein; LDL, low-density lipoprotein.

## Data Availability

The original data used to support the findings of this study are available from the corresponding author upon request.
